# Ascites in Cirrhosis of the Liver

**Published:** 1905-07-08

**Authors:** 


### ASCITES IN CIRRHOSIS OF THE LIVER.

In a clinical lecture on a case of cirrhosis of the liver Dr. Saunby[1] expresses the view that ascites in this condition is due to obstruction of the portal circulation, and that in many cases satisfactory results are obtained by paracentesis. The fluid has usually the characters of a simple transudation and any traces of inflammation discovered post-mortem should be regarded as the result of a complicating, and probably terminal, peritonitis. Of 56 cases in hospital during the last 20 years, Dr. Saunby reports 40 as "recovered," while 16 died in hospital. Many of the cases were admitted for treatment on more than one occasion. As illustrations of the efficacy of tapping the following are quoted. (1) A case tapped four times in five years; no return of fluid after the fourth paracentesis. (2) Patient tapped twice and then lived comfortably for three years when accidentally poisoned. (3) Patient tapped five times between June and October 1901, now reported in good health. Dr. Saunby's views coincide with what was generally accepted doctrine until a few years ago. Since that date an attempt has been made to get rid of the mechanical explanation of ascites and to suggest that the true interpretation of this and of other symptoms in hepatic cirrhosis is to be found in the presence of toxins in the blood, the result of a condition of hepatic inadequacy. Similarly it has been held that true ascites in cirrhosis is a late and, indeed, a terminal event, and that cases in which tapping has been apparently successful in prolonging life have been instances of chronic peritonitis and not of true (non-inflammatory) ascites. Discussing the value of operative measures conducted for the purpose of establishing new vascular routes for the drainage of the peritoneum, Dr. Saunby holds that the risks are too real to justify operation as a routine treatment until, at least, simple tapping has had a fair trial.

[1] Pract., June 1905.

